# Morphometric study of suprascapular notch and scapular dimensions in Ugandan dry scapulae with specific reference to the incidence of completely ossified superior transverse scapular ligament

**DOI:** 10.1186/s12891-020-03769-2

**Published:** 2020-11-10

**Authors:** Adesanya Olamide Adewale, Okeniran Olatayo Segun, Ibe Michael Usman, Ann Lemuel Monima, Eric Simidi Kegoye, Keneth Iceland Kasozi, Halima Nalugo, Fred Ssempijja

**Affiliations:** 1 Department of Anatomy, Faculty of Biomedical Sciences, Kampala International University Western Campus, Ishaka, Bushenyi, Uganda; 2 Department of Physiology, Faculty of Biomedical Sciences, Kampala International University Western Campus, Ishaka, Bushenyi, Uganda; 3 Department of Anatomy, Mbarara University of Science and Technology, Mbarara, Uganda

**Keywords:** Suprascapular notch, Completely ossified superior transverse scapular ligament, Scapular dimensions, Suprascapular nerve entrapment syndrome, Anatomical variation, Ugandan population

## Abstract

**Background:**

Understanding of suprascapular notch (SSN) anatomy and relationship with scapular dimensions are vital in diagnosis, prevention, and assessment of suprascapular nerve entrapment syndrome. The study aimed to assess morphometry of suprascapular notch and scapular dimensions in Ugandan dry scapulae with specific reference to scapulae with completely ossified superior transverse scapular ligaments.

**Methods:**

This was a cross-sectional analytical study conducted on 50 Ugandan dry scapulae. SSN types and prevalence of completely ossified superior transverse scapular ligament among dry scapulae were quantified and compared with previous data. Scapular dimensions were assessed by measuring scapular length (A), scapular width (B), glenoid length (C), and glenoid width (D). One-way ANOVA was used to compare scapular dimensions of scapulae with different SSN types, and Spearman’s correlation coefficient was used to evaluate the correlation coefficient of scapular dimensions amongst groups.

**Results:**

Superior transverse scapular ligament (STSL) was completely ossified in 8% of cases. There was no significant (*P* > 0.05) difference between scapular dimensions of scapulae with completely ossified STSL compared to scapulae with other SSN types. Scapulae with completely ossified STSL showed strong negative (r = − 0.89137, r = − 0.877) correlations for its A, B respectively compared against D, this finding was not true to scapulae of other SSN types. Also, there were strong positive or negative (r > 0.7, r > − 0.7) correlations: for A, types I and III compared to type VI; for B, types I, III compared to VI; for C, type IV and VI; and for D, type III and VI.

**Conclusions:**

The prevalence of completely ossified STSL is moderately high in the Ugandan population. Characteristics of the scapula (scapular dimensions) are not ‘vital’ but rather important or relevant for shoulder pathology with specific reference to suprascapular nerve entrapment syndrome due to completely ossified superior transverse scapular ligaments. Further correlation analyses of scapular dimensions of different SSN types in different populations are important.

**Supplementary Information:**

The online version contains supplementary material available at 10.1186/s12891-020-03769-2.

## Background

The anatomical variations of the suprascapular notch (SSN) are commonly associated with the anomalies of the suprascapular nerve (SN) such as the suprascapular nerve entrapment syndrome. The knowledge of the anatomy and variations of SSN is important for clinicians and surgeons that carry out diagnoses, assessment, management, surgical interventions at the shoulder region. This knowledge is vital in the diagnosis, management, assessment, and prevention of suprascapular nerve entrapment disorder around the shoulder joint [1–3]. The suprascapular notch (SSN) forms a depression on the lateral aspect of the superior border of the scapula, medial to the coracoid process [4], is bridged by the superior transverse scapular ligament (STSL), which may sometimes be completely ossified with a resultant conversion of the notch into a foramen [4]. This foramen serves as a passage to the suprascapular nerve (SN) that supplies sensory branches to the rotator cuff and motor branches to the supraspinatus, and infraspinatus muscles [4, 5], it also provides sensory innervation to the acromioclavicular joint and ligaments, glenohumeral joint, and associated ligaments [6]. Anatomical variations of the SSN were previously classified into six types, by Rengachary et al. in 1979: type I (absent notch or shallow notch), II (shallow V notch), III (U notch), IV (deep V notch), V (partial ossification of superior transverse scapular ligaments) and VI (complete ossification of superior transverse scapular ligaments) based on their morphologic and geometric features [7]. The SSN anatomical variations such as the ossification of the STSL are major risk factors for suprascapular neuropathy due to entrapment of the SN [6–8], a serious issue especially among individuals who are involved in violent overhead activities, such as volleyball, basketball, baseball, tennis players [9–11]. The SN neuropathy associated with anatomical variation of SSN was first described by Kopell and Thompson in 1959, and today approximately 1–2% of all shoulder pains arise from the syndrome with signs and symptoms such as weakness of the arm, inability to properly externally rotate and abduct the arm, and atrophy of the infraspinatus and supraspinatus muscles [9, 11]. Therefore, morphometric anomalies/variations of the SSN can predispose one to an SN entrapment syndrome with the neuropathy being more common among individuals with smaller SSNs such as type III, IV, and VI than individuals with larger SSN types (type I, II, and V) [4, 7, 9, 12]. This is due to dimension and shape factors related to the SSN and suprascapular foramen (SSN morphometric features) [7, 13].

Previously, the clinical value of the morphometric features of SSNs, and the associated classification regarding the anatomical variation of the SSNs have been studied among the different populations such as the American, Greek, Indian, and Kenyan populations [4, 7, 14, 15]. Classifying the SSNs using anatomical variations of the SSN has been found to simplify the clinicians’ work especially when it is incorporated with other methods that could help to link SN entrapment with SSN type in terms of SSN characteristics such as shape and size [14]. The studies showed and re-emphasized the role of anatomically anomalous SSNs such as the completely ossified superior transverse scapular ligament in the emergence of SN entrapment syndrome [4, 7, 14]. Studies have enormously indicated the complete ossification of the STSL (lack of SSN), the formation of the suprascapular foramen, and other morphometric variations of the SSN such as scapulae with type III SSN (scapulae having smaller suprascapular foramina) as the major causes of the SN entrapment syndrome [4, 15].

Data regarding correlation analyses of anthropometric measurements of different SSN types concerning the morphological dimensions of the scapulae are scarce [9, 16], some of the available data have indicated variability in results concerning these studies [9, 16]. While one showed a presence of a relationship between scapular dimensions and morphometry of SSN [9], another recorded an absence of a relationship between scapular dimensions and the morphometric characteristics of the SSN such as shape and type of the SSN [16]. However, both Albino et al. and Polguj et al. were in concurrence on the view that the entrapment syndrome is more likely to be associated with specific notch types (type III and VI) because of their specific features and characteristics such as shape and dimensions of SSN and suprascapular foramen [9, 16]. The relationship between SSN types and basic morphometric scapular dimensions and the clinical value of this relationship in identifying scapulae with completely ossified STSL is largely unknown in various communities of Africa with specific reference to Uganda, this knowledge would be vital for easier diagnosis, assessment, and prevention of shoulder pathologies.

The study aimed to assess the morphometry of the suprascapular notch and scapular dimensions in Ugandan dry scapulae with specific reference to scapulae with completely ossified superior transverse scapular ligaments.

## Methods

### Study design

This was a scientific cross-sectional analytical study. It was conducted at the Museum of the Department of Human Anatomy of Kampala International University, Western Campus located in Western Uganda. The samples were sourced from institutional cadavers of Kampala International University, western campus. Our research was conducted in compliance with the Helsinki Declaration with the formal approval of the Scientific and Ethics review committee of Kampala International University Western Campus, Uganda (Nr.UG-REC-023/202014). A total of 50 dried human scapulae belonging to the anatomical collection in the Anatomy Museum of the Department of Anatomy of the University were included in the study. The fifty dry human scapulae were obtained from adult donors of unknown gender and were randomly selected using a random number algorithm in MS Excel Version 2019 and used as the sample specimens (*n* = 50) for the study. The broken scapulae were excluded from the study.

### Determination of the proportion of suprascapular notch types and scapular dimensions

The shapes of the scapular notches were classified using Renganchary’s system as types I, II, III, IV, V, and VI [7], similar to a previous study by Albino et al. [16]. Representative photographs of the various notch types were taken using a digital camera (Olympus Stylus 600 6MP). The results on different types of SSNs among 50 scapulae were recorded as counts and percentages and these were compared with existing data from previous studies.

The morphological dimensions of scapulae were assessed using previous methods [16, 17] by measuring two distances for each scapular body (scapular length and width): (i) scapular length: the major longitudinal axis of the scapular body, measured from the medial angle to the inferior angle of the scapula (A axis); (ii) scapular width: the major transversal axis of the scapular body, measured from the lowest point of the glenoid to the vertebral border of the scapula at the level of the smooth surface over which the trapezius glides (B axis) [16, 17] (Fig. 3). The morphological dimensions of glenoid fossa were assessed using previous methods [16, 17] by measuring two distances for each glenoid fossa (glenoid length and width): (i) glenoid length: the major longitudinal axis of the glenoid fossa, measured from the supraglenoid tubercle to the lowest point of the glenoid (distance C); (ii) glenoid width: the major transversal axis of the glenoid fossa, measured at its widest distance from the midline of the anterior to the midline of the posterior (distance D) [16, 17] (Fig. 3). All the measurements were performed thrice using a digital Vernier caliper (Mitutoyo 500–153 Absolute Caliper 0-300 mm Range-SPC) and recorded in centimeters.

### Statistical analysis

All statistical analyses were done using GraphPad Prism v.6 and MS Excel Version 2019 and presented in tables and bar graphs. Discrete/categorical variables (types of scapular notches) were reported as percentages and numerical counts; continuous variables (scapular dimensions of each SSN type) were reported using descriptive statistics as mean, median, standard deviation, and range. Ordinary one-way ANOVA (Tukey’s multiple comparisons test) was used to compare the dimensions in different types of notches. Spearman’s rank correlation coefficient was used to evaluate the correlation of dimensions in different types of notches. A value of *p* < 0.05 was considered statistically significant.

## Results

### Prevalence of scapulae with completely ossified STSL is moderate and scapulae with type III SSN are the most prevalent in the Ugandan population

**Fig. 1:**
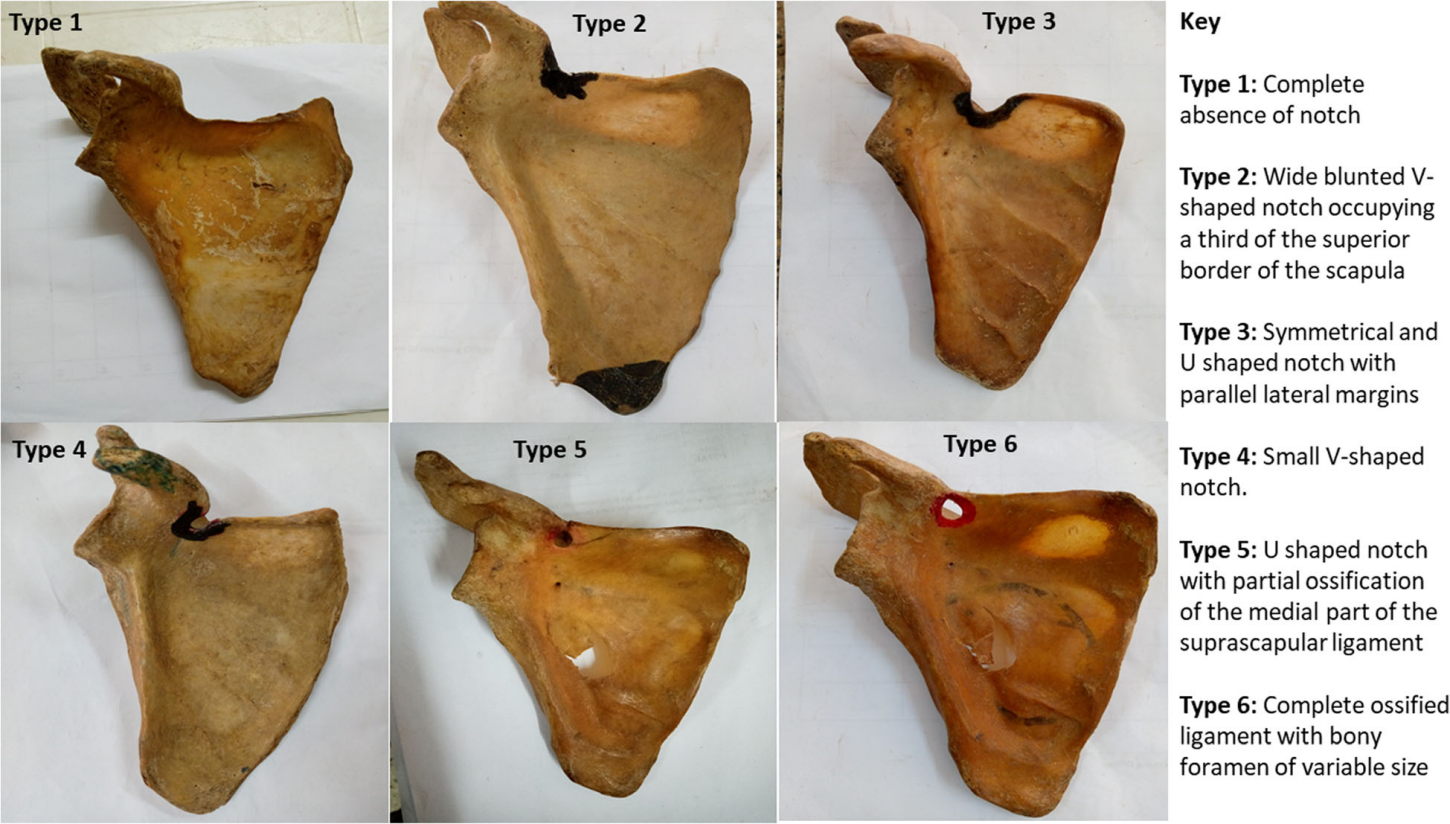
Photographs of the scapulae representing the suprascapular notch types of the study

**Fig. 2:**
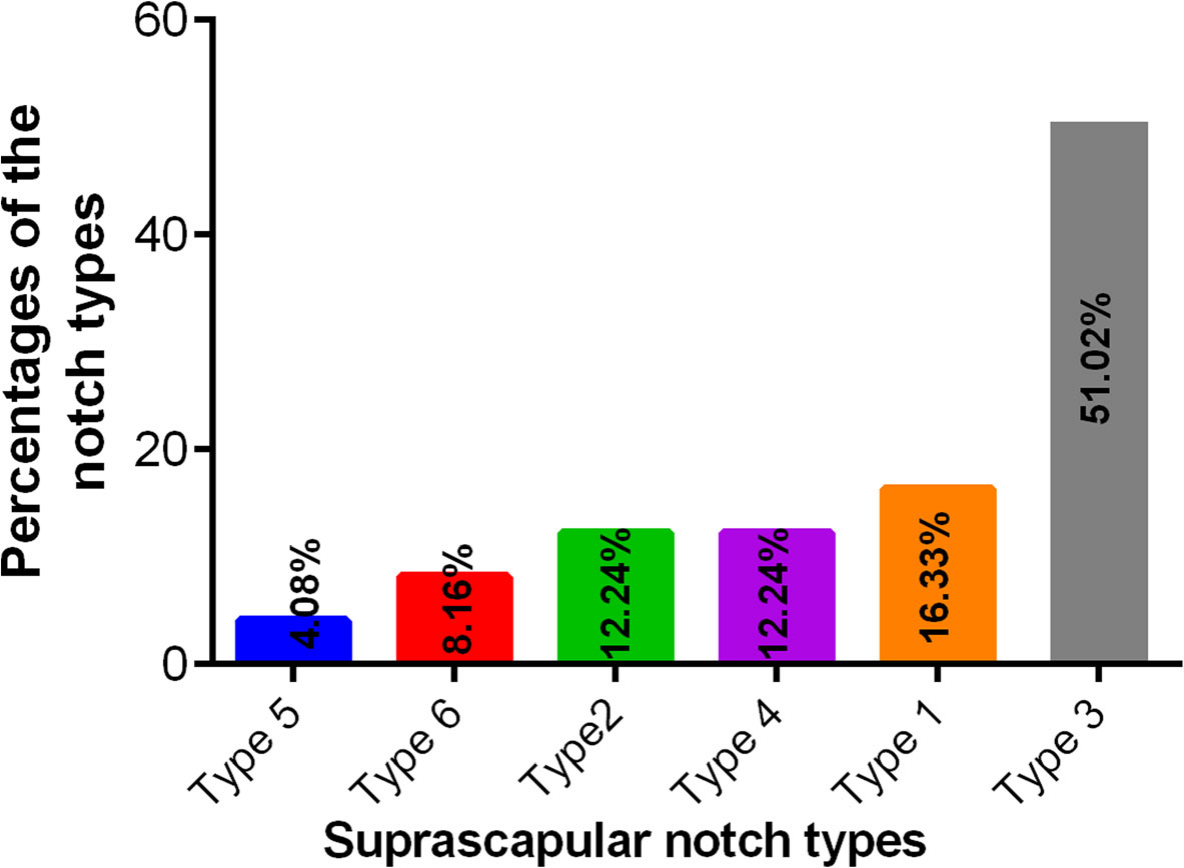
Percentage distribution of the suprascapular notch types

In examining the frequencies and percentages of SSN types, the highest prevalence is of type III (51%) among the whole population. The superior transverse scapular ligament is completely ossified in 8% of cases with an absence of the notch (type VI) (details in Figs. 1, 2**,** and Table 1).

**Table 1:** Comparison of the percentages of the different types of suprascapular notch presented in the literature

Author	Type I	Type II	Type III	Type IV	Type V	Type VI	Population (N)
**Rengachary et al., 1979** [7]	8%	31%	48%	3%	6%	4%	American (211)
**Natsis et al., 2007** [14]	8.3%	42%	42%	7%	0.7%	0%	Greek (423)
**Sinkeet et al., 2010** [15]	22%	21%	29%	6%	19%	3%	Kenyan (138)
**M. Polguj et al, 2011** [9]						7%	Polish (86)
**Albino et al, 2013** [16]	21.4%	19.8%	22.8%	31.1%	10.2%	3.6%	Italian (500)
**Polguj et al., 2013** [11]	24.2%	1.9%	56.2%	4.7	4.7%	0%	Polish (308)
**Kannan, 2014** [4]	20%	10%	52%	4%	4%	10%	Indian (415)
**Our study, 2020**	16.3%	12.2%	51%	12.2%	4.1%	8.2%	Ugandan (50)

*N* Number of the sample

### Scapulae of type IV showed the largest while scapulae of types II and VI SSN had the smallest mean scapular length, glenoid length, and width

**Fig. 3:**
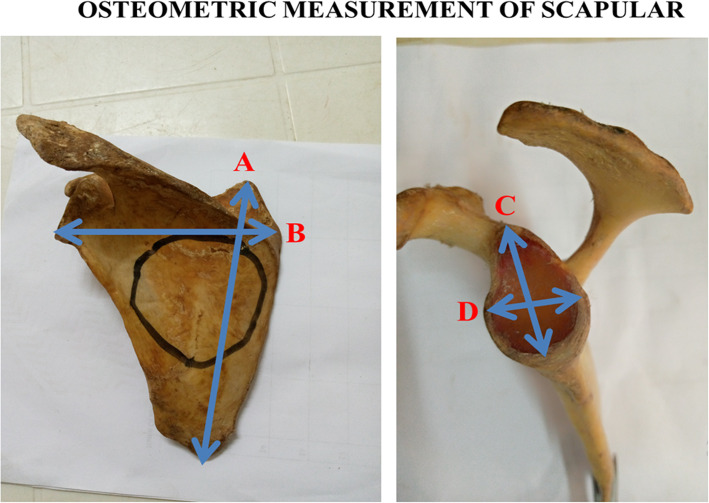
Photograph showing the osteometric measurements of a left scapula. Anterior view: **a** Major longitudinal axis of the scapular body; **b** Major transversal axis of the scapular body. Lateral view: **c** Major longitudinal axis of the glenoid fossa; **d** The major transversal axis of the glenoid fossa

The scapulae with type IV SSN had the largest mean scapular length (15.55 cm), glenoid length (3.817 cm), glenoid width (2.95 cm), scapulae with type VI SSN had the largest scapular width (10.8 cm). Scapulae with type II SSN had the lowest mean scapular length and width (11.9, 8.9 cm respectively); while those with type VI SSN had the lowest mean glenoid length and width (3.325 and 2.250 cm respectively). Descriptive statistics related to the morphometric dimensions of the scapulae of the SSN types are summarized in Fig. 3, and Table 2.

**Table 2:** Descriptive analysis of the morphometric dimensions of scapulae of suprascapular notch types

SSN Type	Mean (cm)	Median (cm)	Standard deviation	Range (cm)
**Type I**				
A	15.14	15.50	1.004	13.50–16.00
B	10.39	11.00	1.233	8.300–11.30
C	3.613	3.650	0.3758	3.000–4.000
D	2.425	2.550	0.2659	2.000–2.600
**Type II**				
A	11.90	11.90	0.2098	11.60–12.20
B	8.817	8.900	1.233	8.300–9.000
C	3.433	3.400	0.1366	3.300–3.600
D	2.283	2.250	0.09832	2.200–2.400
**Type III**				
A	14.37	14.50	0.9320	13.20–15.40
B	9.970	10.30	0.6996	9.000–10.60
C	3.448	3.500	0.2213	3.100–3.700
D	2.735	2.600	0.5449	2.200–3.500
**Type IV**				
A	15.55	15.90	0.6442	14.50–16.00
B	10.78	11.00	0.7885	9.500–11.50
C	3.817	3.900	0.2137	3.500–4.000
D	2.950	2.800	0.5282	2.400–3.600
**Type V**				
A	15.50	15.50	0.7071	15.00–16.00
B	10.45	10.45	0.4950	10.10–10.80
C	3.650	3.650	0.2121	3.500–3.800
D	2.650	2.650	0.2121	2.500–2.800
**Type VI**				
A	13.45	13.20	2.402	11.40–16.00
B	10.10	9.850	1.667	8.700–12.00
C	3.325	3.300	0.1258	3.200–3.500
D	2.275	2.250	0.09574	2.200–2.400

A = the major longitudinal axis of the scapular body; B = the major transversal axis of the scapular body; C = the major longitudinal axis of the glenoid fossa; D = the major transversal axis of the glenoid fossa. *SSN* Ssuprascapular notch

### The morphometric dimensions of scapulae with type VI SSN were not statistically different from those of the other suprascapular notch types

**Fig. 4:**
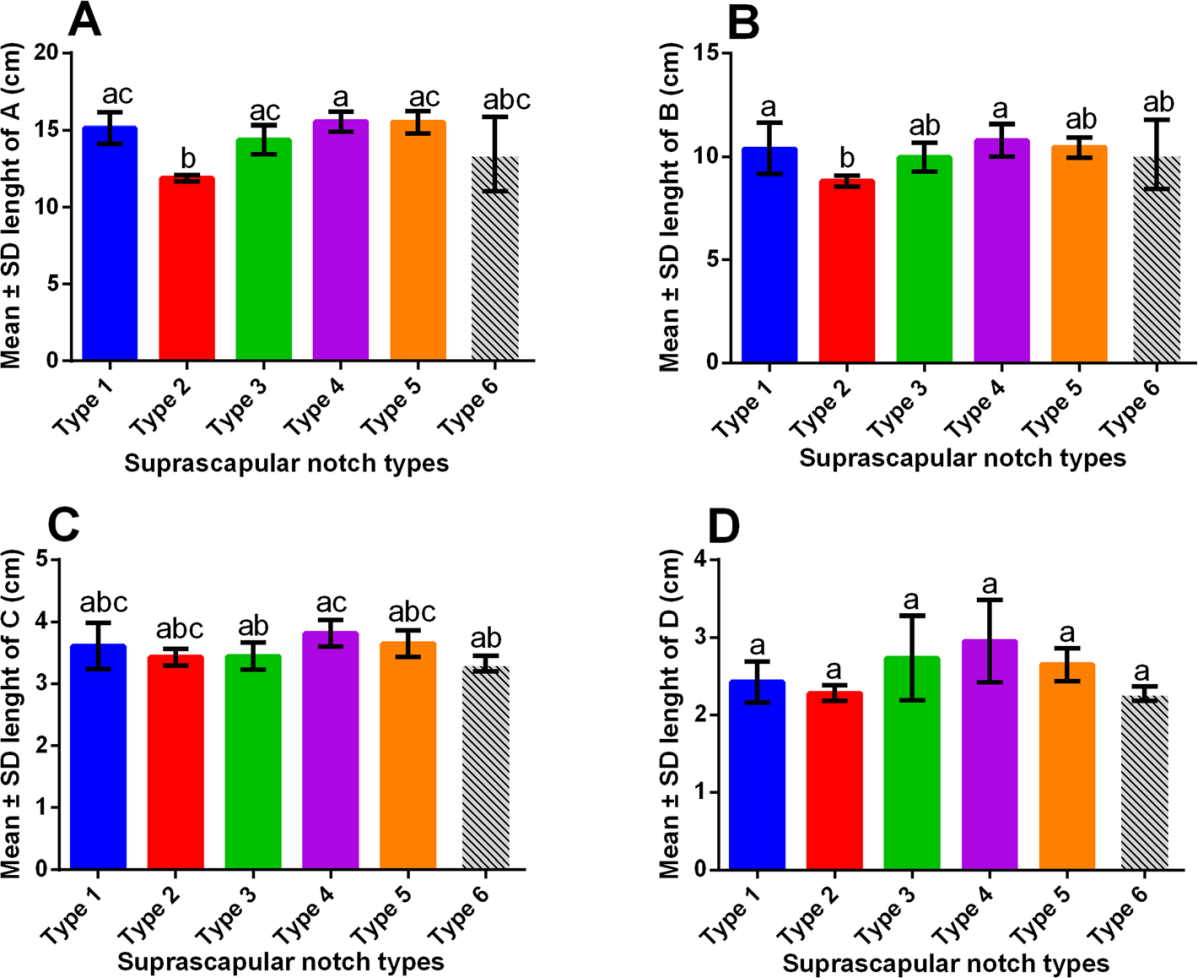
Bar graphs showing the morphometric dimensions of the scapula. **a** The major longitudinal axis of the scapular body; **b** The major transversal axis of the scapular body; **c** The major longitudinal axis of the glenoid fossa; **d** The major transversal axis of the glenoid fossa. Each value is expressed as mean ± S.D. S. D = Standard deviation. **a**, **b**, and **c** represent relationships at *p* < 0.05

The scapular length (A) of type VI SSN was not statistically different (*P* > 0.05) from those of types I, II, III, and V; but was statistically different (*P* = 0.0342) from that of type IV. The scapular width (B) of type VI SSN was not statistically different from the rest of the SSN types. The glenoid length (C) of type VI SSN was not statistically significantly different (*P* > 0.05) from types I, II, III, and V; but was statistically different (*P* = 0.0312) from that of type IV. The glenoid width (D) of type VI SSN was not statistically different from the rest of the SSN types. Details of the multiple comparisons on the morphometric dimensions of scapulae of the SSN types are in Fig. 4 and Additional file 1.

### There were strong positive correlations between the scapular length and width for all the SSN types

For all the SSN types in general, there was a strong positive relationship (r = 0.920547) between A and B; and moderate positive relationships for the remaining correlations between the variables (details in Table 3).

**Table 3:** Correlation coefficient for suprascapular notch and scapular morphometric measurements

	A	B	C	D
**A**	1			
**B**	0.920547	1		
**C**	0.576491	0.674716	1	
**D**	0.430148	0.513304	0.690425	1

A = the major longitudinal axis of the scapular body; B = the major transversal axis of the scapular body; C = the major longitudinal axis of the glenoid fossa; D = the major transversal axis of the glenoid fossa

### There were strong positive correlations between scapular length and width and strongly negative correlations between scapular length, width against glenoid width for scapulae with type VI SSN

In general, there were strong positive correlations (r = 0.7–1) among dimensions A, B, C, and D of types I, III, and IV. In SSN type VI there was a strong positive relationship (r = 0.997069) between A and B axes, strong negative relationships (r = − 0.89137, − 0.877) between A and D, B and D respectively, as well as weak negative correlations (r = − 0.22608, − 0.22243, − 0.20751) between A and C, B and C, C and D respectively (details in Table 4).

**Table 4:** Correlation coefficient of each SSN type of different morphometric dimensions

	A	B	C	D
**Type 1**				
A	1			
B	0.988912	1		
C	0.850258	0.841722	1	
D	0.980342	0.98981	0.768303	1
**Type 2**				
A	1			
B	0.597196	1		
C	0.279145	0.143823	1	
D	−0.29093	0.537122	0.049629	1
**Type 3**				
A	1			
B	0.972381	1		
C	0.692801	0.828927	1	
VD	0.401379	0.599059	0.927954	1
**Type 4**				
A	1			
B	0.970608	1		
C	0.980641	0.98719	1	
D	0.767034	0.881222	0.85935	1
**Type 5**				
A	1			
B	no value	1		
C	no value	no value	1	
D	no value	no value	no value	1
**Type 6**				
A	1			
B	0.997069	1		
C	−0.22608	−0.22243	1	
D	−0.89137	−0.877	−0.20751	1

no value = #DIV/0! error value because the standard deviation of the values equals zero in Type V suprascapular notch. A = the major longitudinal axis of the scapular body; B = the major transversal axis of the scapular body; C = the major longitudinal axis of the glenoid fossa; D = the major transversal axis of the glenoid fossa. *SSN* Suprascapular notch

### There were strongly positive and negative correlations for scapular and glenoid length of type VI SSN compared to the scapulae of other SSN types

There was a strong positive correlation (r = 0.984199) between A of type III and A of type VI, a strong negative relationship (r = − 0.76664) between A of type I and A of type VI. There was a strong positive correlation (r = 0.994792) between B of type III and B of type VI, a strong negative relationship (r = − 0.78853) between B of type I and B of type VI, and a moderate negative relationship (r = − 0.61113) between B of type II and B of type VI. There was a strong positive correlation (r = 0.723339) between C of type IV and C of type VI. There was a moderately positive correlation (r = 0.636364) between D of type I and D of type VI, and a strong negative relationship (r = − 0.72408) between D of type III and D of type VI (details in Table 5).

**Table 5:** Correlation coefficient of each morphometric dimension for scapulae of different suprascapular notch types

	Type 1	Type 2	Type 3	Type 4	Type 5	Type 6
**A**						
Type 1	1					
Type 2	−0.20484	1				
Type 3	−0.08035	−0.25059	1			
Type 4	−0.18342	0.192408	0.294996	1		
Type 5	no value	no value	no value	no value	1	
Type 6	−0.76664	−0.33322	0.984199	−0.05088	no value	1
**B**						
Type 1	1					
Type 2	0.051943	1				
Type 3	−0.03034	−0.4133	1			
Type 4	−0.09387	0.758566	0.035569	1		
Type 5	no value	no value	no value	no value	1	
Type 6	−0.78853	−0.61113	0.994792	−0.21097	no value	1
**C**						
Type 1	1					
Type 2	−0.41302	1				
Type 3	0.455726	0.368522	1			
Type 4	−0.04801	0.593675	−0.15245	1		
Type 5	no value	no value	no value	no value	1	
Type 6	0.319764	0.187317	−0.2163	0.723339	no value	1
**D**						
Type 1	1					
Type 2	−0.116	1				
Type 3	0.252832	−0.5579	1			
Type 4	0.129556	0.94353	−0.45142	1		
Type 5	no value	no value	no value	no value	1	
Type 6	0.636364	−0.09091	−0.72408	0.080013	no value	1

no value = #DIV/0! error value because the standard deviation of the values equals zero in Type V suprascapular notch. A = the major longitudinal axis of the scapular body; B = the major transversal axis of the scapular body; C = the major longitudinal axis of the glenoid fossa; D = the major transversal axis of the glenoid fossa

## Discussion

The scapulae with type III suprascapular notch (SSN) were the most prevalent in our study (51%) and the prevalence of complete ossification of the superior transverse scapular ligament (STSL; type VI SSN) was moderately high (8.2%). These findings concur with those from previous studies in the American, Greek, Kenyan, Polish, Italian, and Indian populations where the values were 22.8–56.2% and 0–10% for types III and VI respectively, Table 1 [4, 7, 9, 11, 14–16]**.** There are dynamic statistics regarding the prevalence of complete ossification of STSL in the current study among the Ugandan population in comparison to other populations i.e. twice the mean prevalence of the previous studies among multiple populations (4%), eight times more than in the Greek population (0%), about twice that of American (4%), Italian (3.6) and Kenyan (3%) populations, and similar to that of the Polish (7%) and Indian (10%) populations Table 1 [4, 7, 9, 11, 14–16]. But despite the above variability regarding the prevalence of the completely ossified STSL in the Ugandan population, the prevalence of completely ossified STSL in our study (8.2%) falls within the range of the previous data in other populations (0–10%) [4, 7, 9, 11, 14–16] and has been cited as one of the major risk factors associated with the suprascapular neuropathy due to SN entrapment [4, 6–8, 14, 15].

The scapulae with completely ossified STSLs (type VI) of our study could not be differentiated from other SSN types using the basic morphometric scapular dimensions (A, B, C, and D) i.e. the dimensions were statistically similar for all the SSN types, except for type IV SSN. Our findings concur with previous findings that showed no significant differences between the scapular dimensions of different SSN types [9, 16]. This is an indication that the morphological dimensions of the scapular body and glenoid fossa are not straight-forward predictors of the occurrence of SN entrapment syndrome from complete ossification of the STSL associated with type VI SSN. It is worthy to note that types VI and IV SSN had statistically different morphological lengths of the scapular body (A) and glenoid fossa (C), this finding could be exploited in medicine in the prediction, diagnosis, and assessment of the SN Entrapment syndrome caused by either type IV or VI but because previous studies have shown both types as being associated with SN Entrapment syndrome [14], and because scapular width (B) and glenoid width (D) were similar for the two types, this finding is of less clinical value about SN neuropathy. Furthermore, our study has shown that based on the morphological dimensions of scapulae (A, B, C, and D), it is generally not possible to differentiate scapulae of type III and IV from those with type I and II SSNs as all have similar scapular dimensions. Because people with a type IV and III SSNs are more predisposed to the SN entrapment syndrome than those with type I and II [7, 13, 15, 16], the study seems to suggest that our dimensions are of less value in the prediction of occurrence, proper diagnosis, and assessment of the SN entrapment syndrome associated with types III and IV SSN, and although our study established significantly different scapular body lengths between types II, III, and IV this finding needs further exploration before we can conclude it as being useful in differentiating scapulae with type II SSN from those associated with predisposition to SN neuropathy (type III and IV).

For all SSN types in general, there is a strong direct connection between A and B axis; the more A increases, the greater the B axis via an exact linear rule, this finding was in tandem with Polguj et al. [9] where the maximal depth (MD) of the SSN correlated with the morphological length of the scapulae [9]. However, most other morphometric measurements in this population indicate a moderate positive linear relationship via a fuzzy-firm linear rule, indicating that it is not very easy to use these relationships to predict the status of these dimensions in the population and that the patient’s scapular dimensions were not related to the characteristics of the SSN such as shape and type of the SSN [16], a picture similar to the study by Polguj et al. [9] where most of the other correlation relationships such as the moderate negative correlation (weak correlations) between the maximal depth (MD) of the SSN and scapular width-length index (WLI) were obtained [9]. In general, the patient’s scapular dimensions were not related to the characteristic of the SSN (type of SSN) and thus the scapular dimensions are not necessary predictors of type of SSN in a given scapula and vice versa.

Considering all the six types of SSN in our study, scapulae with SSN types I, III, and IV have direct relationships between scapular dimensions A, B, C, and D; as one of these dimensions increases, the others also increase linearly. However, this linear relationship is only observed between scapular axes A and B of type VI and concurs with Polguj et al. [9] where a positive correlation between the maximal depth (MD) and the morphological length of the scapulae was seen. The rest of the correlations related to dimensions of SSN type VI are either strongly (A and D; B and D) or weakly negative indicating an inverse correlation also consistent with Polguj et al. who found a negative correlation between the maximal depth (MD) of the SSN and scapular width-length index [9]. This indicates that for type VI SSN, as the A and B axes increases, the D axis tends to decrease through an exact linear rule. This finding indicates that the relationship between scapular dimensions A, B against D can be exploited to find scapulae with SSN type VI and therefore can be exploited for the possible prediction of SN entrapment syndrome associated with it. There are strong (positive or negative) correlations between types I and III from type VI for A-axis; types I, III from VI for B; type IV and VI for the C axis; and type III and VI for the D axis. From the above findings it can be concluded that correlations from all the four morphological lengths of the scapulae (A, B, C, and D): are strong and statistically significant, and therefore can be used to differentiate types I and III from type VI using the A axis B axis can identify types I, III from VI; C axis can be used to identify type IV from VI, and D axis can be used to identify type III from type VI SSN. These correlations show that correlation studies regarding scapular morphological dimensions (scapular length and width; glenoid length and width) are strong and statistically significant in agreement with Polguj et al. [9] but in contradiction with Albino et al. [16] that got weak correlation indices. The strong correlations obtained in our study could be of clinical value in the prediction, diagnosis, and assessment of SN entrapment syndrome associated with type VI SSN as suggested by Polguj et al. [9] using the correlation between maximal depth of the scapular notch with the morphological length of the scapulae [9].

In general, the correlation analyses of our study have shown that the scapular dimensions (scapular length and width; glenoid length and width) might be of value in the diagnosis, assessment, and prevention of SN entrapment syndrome associated with scapulae of type III, IV and VI SSN emphasizing the clinical significance of scapular dimensions in helping clinicians, radiologists, and orthopedic surgeons perform better with minimal complications in agreement with previous findings [1–3].

The current study had some limitations: it did not focus on the gender and age-related differences associated with the morphometric correlation analyses of the scapula, and it employed a low number of specimens for the correlation analyses. In the future, a prospective study will be conducted using a robust sample size to analyze the gender and age-related differences associated with the correlation analyses of scapular dimensions of different SSN types.

## Conclusions

The prevalence of the completely ossified STSL (type VI) in the Ugandan population was moderately high (8.2%) compared to other populations. The current correlation study has shown that the characteristics of the scapula (scapular dimensions) are not ‘vital’ but rather important or relevant for shoulder pathology. Therefore, correlations analyses could be employed in the prediction, diagnosis, prevention, and assessment of suprascapular nerve entrapment syndrome associated with scapulae that have ‘completely ossified’ superior transverse scapular ligaments. Further studies in different populations, on correlation analyses of scapular dimensions for scapulae with different suprascapular notch morphometric characteristics with specific emphasis to scapulae having completely ossified superior transverse scapular ligaments, are necessary.

## Supplementary Information

**Additional file 1:** **Table 6.** Multiple comparisons on the morphometric dimensions of scapulae of the suprascapular notch types.

## Data Availability

Data files can be accessed at https://figshare.com/s/c624cecf6f2d9693165e

## Abbreviations

ANOVA: Analysis of variance
SN: Suprascapular nerve
SSN: Suprascapular notch
STSL: Superior transverse scapular ligament
