# Supplementary material for: Morphometric study of suprascapular notch and scapular dimensions in Ugandan dry scapulae with specific reference to the incidence of completely ossified superior transverse scapular ligament
Source: BMC Musculoskelet Disord. 2020 Nov 10;21:733. doi: 10.1186/s12891-020-03769-2 (PMC7656716; doi:10.1186/s12891-020-03769-2)
Supplement: Supplementary file 1 — Additional file 1: Table 6. Multiple comparisons on the morphometric dimensions of scapulae of the suprascapular notch types. [file 12891_2020_3769_MOESM1_ESM.docx]

**Additional file 1**

**Table 6:** Multiple comparisons on the morphometric dimensions of scapulae of the suprascapular notch types

| Tukey's multiple  comparisons tests | **Mean 1** | **Mean 2** | **Mean Diff.** | **Adjusted P-Value** |
| --- | --- | --- | --- | --- |
| **A (cm)** |  |  |  |  |
| Type 1 vs. Type 2 | 15.14 | 11.90 | 3.238 | < 0.0001 |
| Type 1 vs. Type 3 | 15.14 | 14.37 | 0.7679 | 0.4743 |
| Type 1 vs. Type 4 | 15.14 | 15.55 | -0.4125 | 0.9762 |
| Type 1 vs. Type 5 | 15.14 | 15.50 | -0.3625 | 0.9977 |
| Type 1 vs. Type 6 | 15.14 | 13.45 | 1.688 | 0.1056 |
| Type 2 vs. Type 3 | 11.90 | 14.37 | -2.470 | < 0.0001 |
| Type 2 vs. Type 4 | 11.90 | 15.55 | -3.650 | < 0.0001 |
| Type 2 vs. Type 5 | 11.90 | 15.50 | -3.600 | 0.0015 |
| Type 2 vs. Type 6 | 11.90 | 13.45 | -1.550 | 0.2105 |
| Type 3 vs. Type 4 | 14.37 | 15.55 | -1.180 | 0.1521 |
| Type 3 vs. Type 5 | 14.37 | 15.50 | -1.130 | 0.6796 |
| Type 3 vs. Type 6 | 14.37 | 13.45 | 0.9196 | 0.5799 |
| Type 4 vs. Type 5 | 15.55 | 15.50 | 0.05000 | > 0.9999 |
| Type 4 vs. Type 6 | 15.55 | 13.45 | 2.100 | 0.0342 |
| Type 5 vs. Type 6 | 15.50 | 13.45 | 2.050 | 0.2237 |
|  |  |  |  |  |
| **B (cm)** |  |  |  |  |
| Type 1 vs. Type 2 | 10.39 | 8.817 | 1.571 | 0.0226 |
| Type 1 vs. Type 3 | 10.39 | 9.970 | 0.4179 | 0.8557 |
| Type 1 vs. Type 4 | 10.39 | 10.78 | -0.3958 | 0.9601 |
| Type 1 vs. Type 5 | 10.39 | 10.45 | -0.06250 | > 0.9999 |
| Type 1 vs. Type 6 | 10.39 | 10.10 | 0.2875 | 0.9945 |
| Type 2 vs. Type 3 | 8.817 | 9.970 | -1.153 | 0.0683 |
| Type 2 vs. Type 4 | 8.817 | 10.78 | -1.967 | 0.0047 |
| Type 2 vs. Type 5 | 8.817 | 10.45 | -1.633 | 0.2298 |
| Type 2 vs. Type 6 | 8.817 | 10.10 | -1.283 | 0.2356 |
| Type 3 vs. Type 4 | 9.970 | 10.78 | -0.8138 | 0.3527 |
| Type 3 vs. Type 5 | 9.970 | 10.45 | -0.4804 | 0.9759 |
| Type 3 vs. Type 6 | 9.970 | 10.10 | -0.1304 | 0.9998 |
| Type 4 vs. Type 5 | 10.78 | 10.45 | 0.3333 | 0.9972 |
| Type 4 vs. Type 6 | 10.78 | 10.10 | 0.6833 | 0.8345 |
| Type 5 vs. Type 6 | 10.45 | 10.10 | 0.3500 | 0.9973 |
|  |  |  |  |  |
| C **(cm)** |  |  |  |  |
| Type 1 vs. Type 2 | 3.613 | 3.433 | 0.1792 | 0.7377 |
| Type 1 vs. Type 3 | 3.613 | 3.448 | 0.1647 | 0.5581 |
| Type 1 vs. Type 4 | 3.613 | 3.817 | -0.2042 | 0.6195 |
| Type 1 vs. Type 5 | 3.613 | 3.650 | -0.03750 | > 0.9999 |
| Type 1 vs. Type 6 | 3.613 | 3.325 | 0.2875 | 0.3844 |
| Type 2 vs. Type 3 | 3.433 | 3.448 | -0.01449 | > 0.9999 |
| Type 2 vs. Type 4 | 3.433 | 3.817 | -0.3833 | 0.0832 |
| Type 2 vs. Type 5 | 3.433 | 3.650 | -0.2167 | 0.8767 |
| Type 2 vs. Type 6 | 3.433 | 3.325 | 0.1083 | 0.9811 |
| Type 3 vs. Type 4 | 3.448 | 3.817 | -0.3688 | 0.0196 |
| Type 3 vs. Type 5 | 3.448 | 3.650 | -0.2022 | 0.8610 |
| Type 3 vs. Type 6 | 3.448 | 3.325 | 0.1228 | 0.9326 |
| Type 4 vs. Type 5 | 3.817 | 3.650 | 0.1667 | 0.9561 |
| Type 4 vs. Type 6 | 3.817 | 3.325 | 0.4917 | 0.0312 |
| Type 5 vs. Type 6 | 3.650 | 3.325 | 0.3250 | 0.6268 |
|  |  |  |  |  |
| **D (cm)** |  |  |  |  |
| Type 1 vs. Type 2 | 2.425 | 2.283 | 0.1417 | 0.9913 |
| Type 1 vs. Type 3 | 2.425 | 2.735 | -0.3098 | 0.5436 |
| Type 1 vs. Type 4 | 2.425 | 2.950 | -0.5250 | 0.2676 |
| Type 1 vs. Type 5 | 2.425 | 2.650 | -0.2250 | 0.9874 |
| Type 1 vs. Type 6 | 2.425 | 2.275 | 0.1500 | 0.9936 |
| Type 2 vs. Type 3 | 2.283 | 2.735 | -0.4514 | 0.2546 |
| Type 2 vs. Type 4 | 2.283 | 2.950 | -0.6667 | 0.1216 |
| Type 2 vs. Type 5 | 2.283 | 2.650 | -0.3667 | 0.9129 |
| Type 2 vs. Type 6 | 2.283 | 2.275 | 0.008333 | > 0.9999 |
| Type 3 vs. Type 4 | 2.735 | 2.950 | -0.2152 | 0.8968 |
| Type 3 vs. Type 5 | 2.735 | 2.650 | 0.08478 | 0.9998 |
| Type 3 vs. Type 6 | 2.735 | 2.275 | 0.4598 | 0.4136 |
| Type 4 vs. Type 5 | 2.950 | 2.650 | 0.3000 | 0.9614 |
| Type 4 vs. Type 6 | 2.950 | 2.275 | 0.6750 | 0.1985 |
| Type 5 vs. Type 6 | 2.650 | 2.275 | 0.3750 | 0.9245 |

A= the major longitudinal axis of the scapular body; B= the major transversal axis of the scapular body; C= the major longitudinal axis of the glenoid fossa; D= the major transversal axis of the glenoid fossa.
